# Primary cemented total hip arthroplasty: 10 years follow-up

**DOI:** 10.4103/0019-5413.65152

**Published:** 2010

**Authors:** Rajendra Nath, Anil Kumar Gupta, Unmesh Chakravarty, Rohit Nath

**Affiliations:** Department of Orthopedic Surgery, GSVM Medical College, Kanpur-208 002, India

**Keywords:** Total hip arthroplasty, cemented hip arthroplasty, Harris hip score

## Abstract

**Background:**

Primary cemented total hip arthroplasty is a procedure for non-traumatic and traumatic affections of the hip. Long term follow-up is required to assess the longevity of the implant and establish the procedure. Indo-Asian literature on long term result of total hip arthroplasty is sparse. We present a 10-year follow-up of our patients of primary cemented total hip arthroplasty.

**Materials and Methods:**

We operated 31 hips in 30 patients with primary cemented total hip arthroplasty. We followed the cases for a minimum period of 10 years with a mean follow-up period of 12.7 years. The mean age of the patients was 60.7 years (range 37–82 yrs) male to female ratio was 2:1. The clinical diagnoses included - avascular necrosis of femoral head (n=15), sero positive rheumatoid arthritis (n=5), seronegative spondylo-arthropathy (n=4), neglected femoral neck fractures (n=3), healed tubercular arthritis (n=2) and post traumatic osteoarthritis of hip (n=2). The prostheses used were cemented Charnley’s total hip (n=12) and cemented modular prosthesis (n=19). The results were assessed according to Harris hip score and radiographs taken at yearly intervals.

**Results:**

The mean follow-up is 12.7 yrs (range 11-16 yrs) Results in all operated patients showed marked improvement in Harris hip score from preoperative mean 29.2 to 79.9 at 10 years or more followup. However, the non-inflammatory group showed more sustained long term improvement as compared to the inflammatory group, as revealed by the Harris hip score. Mean blood loss was 450ml (±3.7 ml), mean transfusion rate was 1.2 units (±.3). The complications were hypotension (n=7), shortening >1.5 cm (n=9), superficial infection (n=2) and malposition of prosthesis (n=1).

**Conclusion:**

The needs of Indian Asian patients, vary from what is discussed in literature. The pain tolerance is greater than western population and financial constraints are high. Thus revision surgery among Indian-Asian patients is less compared to western yard sticks.

## INTRODUCTION

Forty four years after Sir John Charnley^1^ conducted the first cemented total hip arthroplasty, science has come up with fascinating advances and promising breakthroughs aimed at increasing the longevity of the implant and maximizing the function of a joint. While short term results with many designs have been highly promising, only a few Asian–Indian studies have a successful long term follow-up.^2^ Keeping in mind these two conflicting opinions, we present a systematic review of our cases and long term follow-up of our total hip arthroplasty cases.

## MATERIALS AND METHODS

31 consecutive hips in 30 patients undergoing cemented total hip arthroplasty between March 1995 to January 1998 were included in study. There were 10 females and 20 male patients. In one male patient, bilateral total hip arthroplasty was done in two sittings. The mean age of patients was 60.7 years (range 37 to 82 years). There were 15 cases of avascular necrosis of the femoral head, five cases of sero-positive rheumatoid arthritis, four cases of sero negative spondylo arthropathy, two cases of healed tuberculosis of the hip joint, two cases of post traumatic osteoarthritis of hip and three cases of neglected femoral neck fractures with degenerative changes. Only those cases were included in study in which a minimum follow up of 10 years was available.

In our series, primary osteoarthritis was not reported. There were 12 cemented Charnley’s total hips and 19 cemented modular total hips (INOR, Mumbai). Cemented modular total arthroplasty was performed in those patients who could afford implant. In the other patients, Charnley type of cemented total hip arthroplasty was done; though modularity helped in setting the right offset, this has inherent cost implications.

After thorough history taking, clinical evaluation, investigations and necessary pre-operative surgical planning was done.

Standard radiological assessment was done using radiographs of pelvis with both hips in anteroposterior (AP) and cross leg lateral views with magnification markers. The same X-rays were used for overlay templating using Capello’s technique.^3^ The center of acetabulum was marked with the help of acetabular template. Center of femoral head and size of prosthesis and neck cut were marked using femoral template. Minor adjustments in offset were made at the time of surgery either by using the implant of different off set or a modular component. Fitting of medullary femoral component in lateral view was also preoperatively planned.

### Operative procedure

The epidural anesthesia and lateral position was used. Posterior Moore’s approach was used in all the patients. The attachment of gluteus maximus on the back of trochanter was partially removed, if it was felt that the hip was too tight.

After capsulotomy, the hip joint was dislocated posteriorly. The femoral neck was osteotomized at the predetermined level as per preoperative planning. Acetabular reaming was done up to the level when vascular bed was achieved. Cup size was determined by trial cup. Appropriately sized cup was placed using hand mixed cement. The femoral canal was prepared using successively increasing sized rasps and the final size was noted. Trial stem was tested along with modular head and neck. Necessary modification in the sizing was done. Trial components were removed and appropriate sized femoral component was inserted using cement gun, cement restrictor and pressuriser. The joint was reduced by manipulation and the wound was closed in layers. Suction drain was used in all the patients. The operated limb was kept on a pillow without any splintage. Active quadriceps exercises started next day and patient was allowed to stand and walk on the third day with the help of a walking frame. The patients were assessed at one week, two weeks, one month, six months and then at yearly intervals for pain, range of movement and function. The radiological assessment was done in the immediate postoperative period, at six months and then at yearly intervals. Loosening was assessed using Gruen’s^4^ three zones for acetabulum and Delee and Charnley’s^5^ seven zones for the femoral component. Acetabular cup wear was measured on AP radiographs as distance between acetabular cement line and metallic part of head of femur. Protrusio was measured by distance between cup and ilioischial line. Functional assessment was done by Harris Hip score system.^6^ The pre-operative, immediate postoperative and long term complications were recorded.

## RESULTS

The mean follow-up was 12.7 years (range 11-16 years). The male to female ratio was 2:1. The mean age was 60.7 years (range 37-82 years). The mean blood loss was 450 ml (+3.7 ml), mean transfusion rate was 1.2 units (+0.3 unit). Results in all operated patients showed marked improvement in Harris hip score from preoperative mean 29.2 to 79.9 at 10 years or more follow-up. The most common early complication was hypotension (n=7) and late complication was shortening of more than 1.5 cm (n=9).

The comparison of the pre and postoperative clinical scores and their statistical analysis using the paired student’s t-test are shown in Table 1. The mean improvement in pain score was (it was done according to pain component of Harris hip scoring system) from 7.06 to 39.6 at one year which fell down to 35.71 at 10 years. Patients with AVN showed preoperative pain score to be 6.92 and range of motion score was 1 which improved to 39.5 and 3.95 successively at 10 years followup.

**Table 1:** Comparison of preoperative and postoperative clinical scores

Score	Cases	Pre op mean	Mean at 10 years	Pre op ROM score	ROM score at 1 yr	ROM score at 10 yrs	Pre op pain score	Pain score at 1 yr	Pain score at 10 yrs
Overall Harris score	31	29.2	79.9	0.88	3.79	3.55	7.06	39.6	35.71
A.V.N.	15	31.2	85.7	1	4	3.95	6.92	41.07	39.5
F.N.F.	3	42	81.3	0.92	3.91	3.88	7.5	37.5	36.8
O.A+T.B.	4	29.2	85.5	0.5	3.75	3.55	7.1	38.5	38.3
R.A.	5	26	78.8	1	4	3.1	6.66	33.33	27.41
S.N.S.A.	4	15.8	52.8	0.5	3	2.43	5	35	28.5

A.V.N - Avascular necrosis, F.N.F - Fracture neck femur, R.A - Rheumatoid arthritis, S.N.S.A - Sero negative spondylo arthropathy, O.A - Osteoarthritis, T.B - Tuberculosis, R.O.M - Range of movement.

The radiological assessment was done at 10 years. The magnitude of wear was assessed by the simpler methods adopted by Pollock *et al*.^7^ and Mc Calden *et al*.^8^

**Figure 1:**
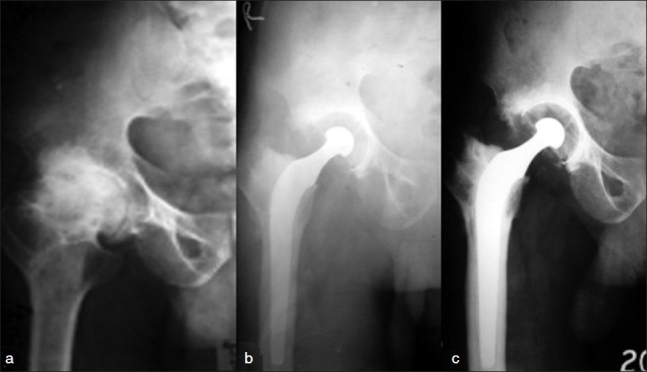
X-ray anteroposterior view of hip joint showing (a) AVN in a case of fracture neck femur treated with Mc Murrays osteotomy. (b) Cemented Charnley’s total hip arthroplasty was done. (c) Acetabular and femoral component cement loosening seen at 10 years followup.

Radiological finding seen in acetabulum at 10 years were cup cement line <1 mm (n=26), cup cement line >1 mm (n=5), protrusion (n=3) and wear of cup (n=1) [Figure 1a‐c]. On femoral side, stem cement radiolucency <1mm (n=23), stem cement radiolucency > 1 mm (n=8), calcar resorption (n=6), migration into varus (n=5), femoral subsidence (n=3) and cyst formation (n=2).

We noted two intraoperative complications in the form of periprosthetic fractures: the first, in a case of AVN with prolonged steroid usage and the second in a case of rheumatoid arthritis. The fractures were managed by encirclage wiring followed by cemented prosthesis placement [Figure 2a‐e].

**Figure 2a:**
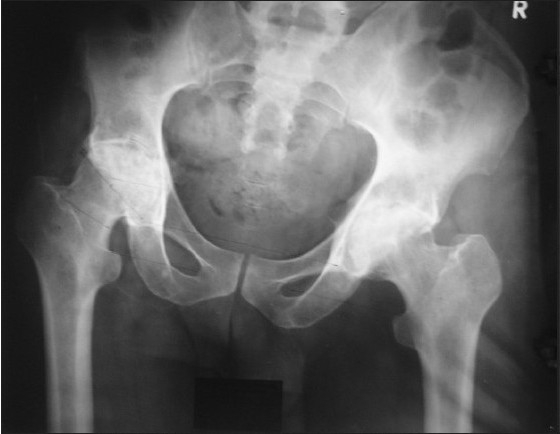
Pre operative X-ray pelvis with both hips showing bilateral femoral head destruction in an inflammatory arthropathy

**Figure 2b:**
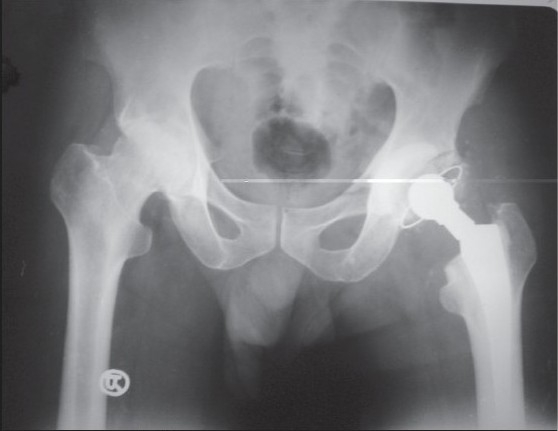
X-ray pelvis with both hips showing stage 1 – Muller

**Figure 2c:**
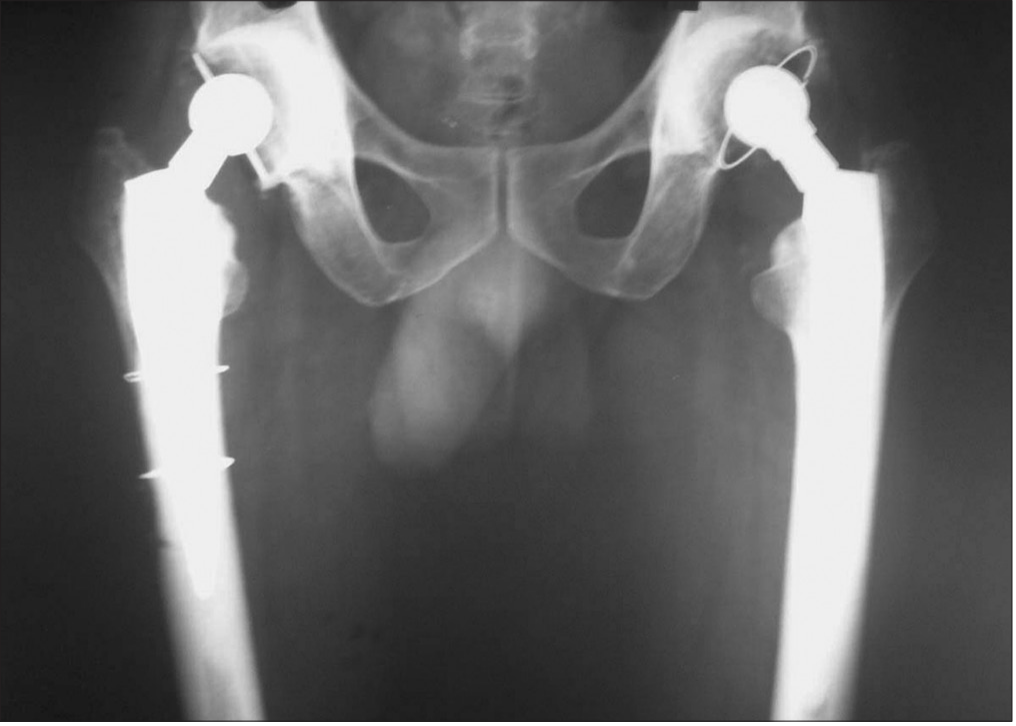
X-ray pelvis with both hips of the same patient, 6 months after the Muller cemented total hip arthroplasty of right hip. The patient sustained intra-operative periprosthetic fracture which was treated with encirclage

**Figure 2d:**
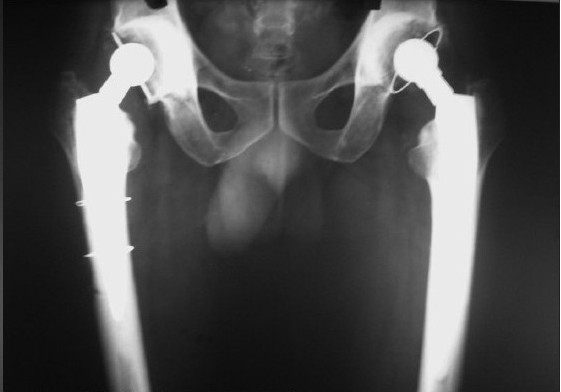
X-ray pelvis with both hips of the same patient showing minimal shortening and subsidence at 11 years follow up

**Figure 2e:**
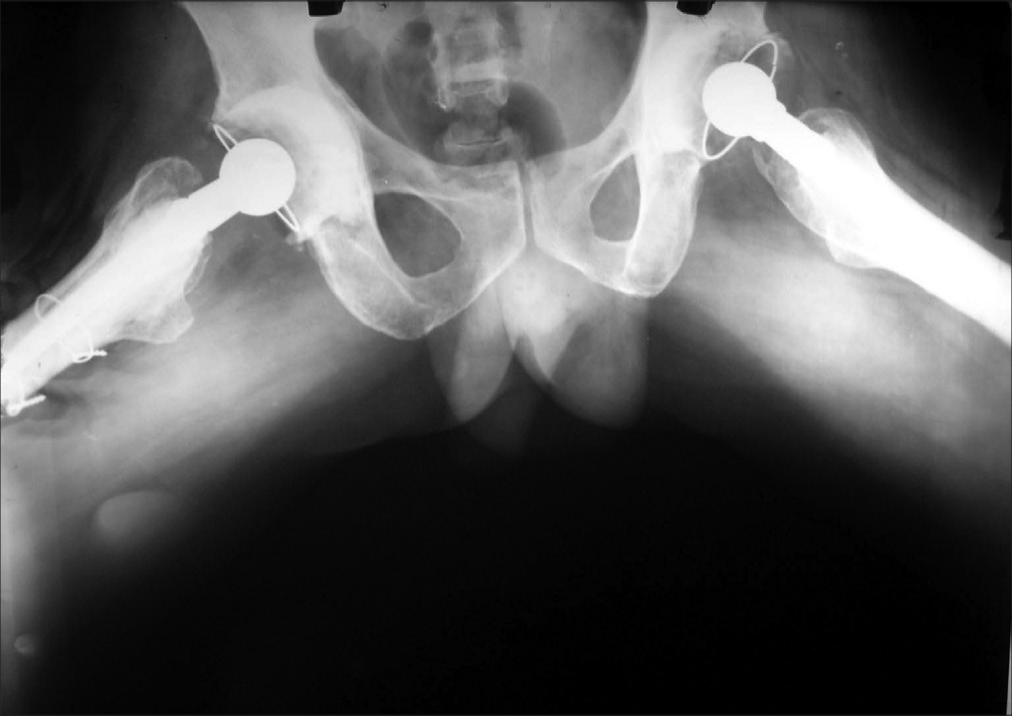
X-ray pelvis with both hips (frog lateral view) showing good range of movement at 11 years followup

There were three cases of peroperative cementing failure. In two cases the cement hardened before the femoral component was fully seated and in one case femoral component was fully seated but was loose. In the first two cases the femur had to be osteotomized followed by canal clearance and encirclage wiring of the fragment. The revision with fresh cement and the same femoral component was done successfully. In the third case the femoral component was removed, cement clearance was done and it was revised. Though the removal of cement led to perforation of the anterior femoral cortex. There was no significant difference in outcome except weight bearing was delayed.

## DISCUSSION

The study aims at evaluating the long term outcome of primary cemented total hip arthroplasty over a period of more than 10 years. Though most short term results seem to be impressive both for the surgeon and the patient, as he improves dramatically from a crippling disease, it is the long term follow-up which actually tells about the longevity, cost effectiveness, strength and weakness of the surgery performed.

We observed the mean improvement in pain score from 7.06 to 39.6 at one year which fell down to 35.71 at 10 years. Though the maximum improvement in pain score and maintenance at 10 years was seen with the osteoarthritis and avascular necrosis group, the maximum improvement was seen in the tubercular hip group and the minimum improvement in the rheumatoid arthritis group. The improvement in the score was irrespective of the preoperative pain score and was dependent more on the type of disease. Maximum improvement was seen in the non inflammatory, non arthritic group while the inflammatory and collagen vascular diseases had less improvement in pain score as well as late increase in pain. These findings suggest that in the inflammatory and collagen vascular diseases, replacing the damaged articular surfaces alone does not reduce pain as much as in the other non inflammatory groups. Probably the constant inflammation around the joint capsule and adjacent soft tissues mimics articular pain. Studies by Amstutz and Sakai^9^ also show good relief of pain in single ankylosed hip but less as compared to the non inflammatory groups.

In our series, primary osteoarthritis has not been reported. As compared to Europe and North America, the Indian subcontinent has very less incidence of primary osteoarthritis of hip. The possible explanation of this can be Indian population use squatting and cross leg sitting and kneel down position during activities of daily living. These postures of hip pass through extreme range of movements and by this process, the nutrition of cartilage is fulfilled in all sectors.

The mean preoperative range of movement score as per the Harris hip score was 0.88 which improved to 3.79 at one year and went down to 3.55 at 10 years. The collagen vascular diseases including rheumatoid arthritis (n=5) and sero negetive spondyloarthropathy (n=4) showed the maximum deterioration in range of movement from 4 to 3.1 and 3 to 2.43 respectively despite good control of the disease with disease modifying drugs and analgesics, but the range of movement score in the monoarticular group (n=22) comprising of avascular necrosis, fracture neck femur, osteoarthritis and tuberculosis showed a marginal deterioration. These facts point to the fact that despite initial improvement there was deterioration when an ongoing inflammation was around or the nearby joints were involved. Siwach *et al*,^10^ reported in series of 100 THA cases, a mean Harris hip score of 44 (range 30-50) preoperatively which improved to 83.5 (range 60-96) post operatively.

The improvement in the overall mean Harris hip score was from 29.2 to 79.9 at ten years with a statistically significant p value of less than 0.001. The maximum postoperative scores were seen with avascular necrosis, osteoarthritis, tubercular hip and fracture neck femur. Studies by Young-Hoo Kim^11^ have shown improvements in Harris score of more than 90 points in cases of avascular necrosis. In recent studies by Blomfeldt *et al*,^12^ it has been seen that in cases of displaced fracture neck femur treated with total hip replacement the rate of complication was 4% as compared to 42% with internal fixation (*P*< 0.001). Also rates of re-operations were 4% in total hip replacement group as compared to 47% in internal fixation group.

The maximum improvement was seen in healed tuberculosis cases which were treated for >12 months with antituberculosis drugs and followed for >5 years, from a score of 15.5 to 87.5. The minimum scores were seen in rheumatoid arthritis and ankylosing spondylitits at 10 years follow up, an improvement of 26 to 78.8 and 15.8 to 52.8 respectively. These scores are suggestive of the final score being influenced by the initial Harris hip score, except for tuberculosis. In tuberculosis the two patients under study were cases of old healed tuberculosis, with a painful fibrous ankylosis of the hip. Relieving the patients off the fibrous ankylosis immediately improved the pain, range of movement and in turn the overall function and quality of life. Since there were no other joint involvement and ongoing inflammatory disorder these patients continued to have excellent relief and improvement in function. Young-Hoo Kim *et al*.,^11^ had performed his study on 170 total hips of which 47 were cemented and had also shown an overall improvement in Harris hip score from 50 to 89 at more than 10 years follow-up. Eight (17%) of forty-seven hips with cemented or hybrid fixation and eighteen (15%) of 123 hips with cementless fixation had revision of both components because of aseptic loosening and/or osteolysis. The mean rate of linear wear of the polyethylene was 0.25 mm per year in the cemented or hybrid group and 0.29 mm per year in the cementless group. The prevalence of osteolysis was 53% (twenty-five of forty-seven hips) in the cemented or hybrid group and 59% (seventy-two of 123 hips) in the cementless group.

The commonest complication in our series was of blood loss and peroperative hypotension. There were 10 cases with blood loss of more than 500 ml and seven cases of hypotension, none lasting more than two hours after blood and fluid replenishment. Of the seven cases of hypotension five had been due to direct blood loss. The other two cases occurred before cementing and were not associated with signs of hypoxemia or respiratory distress and were probably due to anesthetic drugs and none could be specifically pointed out to a particular cause. This is also possibly attributed to our elderly and uncompensated patients. There were no cases of embolism, cardiac arrest or death in our series. This is probably explained by the use of continuous suction in the femoral canal during cementing which has been shown to be associated with lower incidence of fat embolism.^13^

Scott *et al*,^14^ studied 38 cases of peri-prosthetic fractures and found 18 cases were intraoperative and occurred during reaming of the canal, seating of the femoral stem or manipulating the femur. Bryan *et al*,^15^ treated 116 peri prosthetic fractures by revision of femoral stem.In our series, intraoperative periprosthetic fractures occurred in two cases. The fractures united even with encirclage wiring though their final outcomes showed less functional improvement and range of movement. Pellicci *et al*,^16^ studied the rare complication of canal perforation in 12 patients and also found female sex, osteoporosis, previous fracture and previous surgery as predisposing factors. All but two of his subjects were asymptomatic after an average follow-up of five years and had been treated with protected weight bearing for six weeks. Roentgenographic studies showed that penetration of the femoral shaft does not appear to seriously compromise the fixation of the femoral component. Studies by Poss *et al*,^17^ also show patients with rheumatoid arthritis have a definite risk of intraoperative fracture.

There were two cases of superficial infection which were identified in the second week of surgery. They were managed by regular dressing of the wound and antibiotics. Charnley and Cupric^1^ observed superficial infection even after strict maintenance of asepsis in 6.6% cases. Brady *et al*,^18^ also reported a rate of 1% infection with air enclosures but without use of any antibiotics.

There were five cases where the femoral component was in varus. Three of them showed an initial varus malalignment, followed by calcar resorption and implant subsidence. The other two were associated with only calcar resorption. The two cases of late varus migration of prosthesis were not associated with any signs of inflammation or tumor indicating some other mechanism affecting the prosthesis locally. Similar findings were seen by Harris *et al*,^19^ where they found four cases of localized bone resorption in the calcar area without any signs of inflammation.

Shortening reported in our cases could be avoided by thorough preoperative planning and it’s execution. It may be because of lack of awareness in early stage of study rather than type of implants used. However, shortening caused by subsidence is a late phenomenon.

Financial considerations for revision are primary deciding factors in rural Indian setup rather than need. The tolerance of rural patients to pain is an enigma to treating surgeons. The pain and discomfort caused by loosening is a matter of personal endurance. They prefer daily oral analgesic rather than revision. However, none of the patients complained of severe pain. We are of the opinion that the requirement of revision is multifactorial. We shall like to draw attention to the fact that all our patients were ambulatory and discomfort level was within limits of tolerance. We cannot ape western yardsticks for revisions as our circumstances different.
